# Association of ATM Gene Polymorphism with PTC Metastasis in Female Patients

**DOI:** 10.1155/2014/370825

**Published:** 2014-10-16

**Authors:** Yulu Gu, Xiaoli Liu, Yaqin Yu, Jieping Shi, Lizhe Ai, Hui Sun, Joseph Sam Kanu, Chong Wang, Yawen Liu

**Affiliations:** ^1^Department of Epidemiology and Biostatistics, School of Public Health, Jilin University, Changchun 130021, China; ^2^Jilin Provincial Key Laboratory of Molecular Epidemiology, School of Public Health, Jilin University, Changchun 130021, China; ^3^Jilin Provincial Key Laboratory of Surgical Translational Medicine, Department of Thyroid and Parathyroid Surgery, China-Japan Union Hospital, Jilin University, Changchun 130033, China

## Abstract

Ataxia telangiectasia mutated (ATM) gene is critical in the process of recognizing and repairing DNA lesions and is related to invasion and metastasis of malignancy. The incidence rate of papillary thyroid cancer (PTC) has increased for several decades and is higher in females than males. In this study, we want to investigate whether ATM polymorphisms are associated with gender-specific metastasis of PTC. 358 PTC patients in Northern China, including 109 males and 249 females, were included in our study. Four ATM single nucleotide polymorphisms (SNPs) were genotyped using Matrix-Assisted Laser Desorption/Ionization Time of Flight Mass Spectrometry (MALDI-TOF-MS). Association between genotypes and the gender-specific risk of metastasis was assessed by odds ratios (OR) and 95% confidence intervals (CI) under the unconditional logistic regression analysis. Significant associations were observed between rs189037 and metastasis of PTC in females under different models of inheritance (codominant model: OR = 0.15, 95% CI 0.04–0.56, *P* = 0.01 for GA versus GG and OR = 0.08, 95% CI 0.01–0.74, *P* = 0.03 for AA versus GG, resp.; dominant model: OR = 0.49, 95% CI 0.25–0.98, *P* = 0.04; overdominant model: OR = 0.47, 95% CI 0.25–0.89, *P* = 0.02). However, no association remained significant after Bonferroni correction. Our findings suggest a possible association between ATM rs189037 polymorphisms and metastasis in female PTCs.

## 1. Introduction

Papillary thyroid cancer (PTC) accounts for approximately 90% of all histologic types of thyroid cancers, and it is also the most common endocrine malignancy with an increasing incidence over the past several decades [1–3]. Despite improved medical techniques, such as diagnosis and reporting, other accountable reasons remain to be discovered [4]. Risks of PTC comprise genetic and environmental factors [5]. A recent study found that individuals with family history of thyroid cancer, especially in siblings, are highly predisposed to PTC [6]. In addition, female and ionizing radiation (IR) during the period of childhood and adolescents are certain risk factors of PTC [7–9]. In normal conditions, incidence rate of PTC in females is more than males, and the female-to-male rate ratio is about three or more. PTC patients generally have a good prognosis, with the overall 5-year and 10-year relative survival rate being about 95% to 97% and 93%, respectively [10–13]. Clinicopathological features that influence prognosis of PTC include age at diagnosis, primary tumor size, local and extrathyroidal invasion, and metastasis [1, 14]. Studies have identified single nucleotide polymorphism (SNP) in several genes sensitive to PTC, such as FOXE1, BRAF V600E, and Fas gene [15–17]. A genome-wide association study (GWAS) also found that variants on rs965513 and rs944289, which are adjoined by FOXE1 and NKX2-1, are associated with increased risk of PTC [18].

In this study, the gene we were interested in was ataxia telangiectasia mutated (ATM) for its critical function in the process of recognizing and repairing DNA lesions of its coding protein kinase. ATM gene located on chromosome 11q22-23 spanning over 160 kb of genomic DNA and produces an approximately 350 kDa protein that plays a key role in DNA damage response, especially for double-strand break [19]. ATM belongs to the phosphoinositide 3-kinase (PI3-K) family and exists in the form of dimer or multimer [20]. Once cells received radiation, ATM dimer will be dissociated and so activated, followed by a variety of downstream proteins phosphorylation [21, 22]. In the human autosomal recessive disorder ataxia telangiectasia, ATM gene is mutated, resulting in genome instability, immunodeficiency, hypersensitive to IR, and cancer predisposition [23, 24]. Since Savitsky et al. first named the gene, ATM has been widely studied not only about the mechanism and pathway it functions, but also about its association with diseases, like variety of cancers [20]. Malignancies that have been reported to be associated with specific ATM alleles mutation include breast, lung, thyroid, prostate cancer, and chronic lymphocytic leukemia [25–29]. Studies previously suggested that low expression of ATM was related to poor differentiation of oral squamous cell carcinoma and gastric cancer and involved in lymph-node metastasis [30, 31]. A recent study also demonstrated that ATM facilitated invasion and lymph-node metastasis of breast cancer through the ATM-Snail pathway [32]. Similar situation was found in neuroendocrine tumor in which ATM was downregulated in metastatic patients compared with nonmetastatic ones [33]. These findings suggest that ATM gene may be a biomarker for diagnosis and prognosis in cancers.

More than 10% of PTC patients end up dead and considerable percentage of the cancers in these patients metastasize [34, 35]. In this study, we want to investigate whether the presence of the ATM gene polymorphisms indicates metastasis of PTCs.

## 2. Materials and Methods

### 2.1. Patients and Samples

A total of 358 PTC patients undergoing surgery, including 109 males and 249 females, recruited from the China-Japan Union Hospital of Jilin University in Changchun, Jilin province, China, between January 2010 and April 2012, were enrolled in our study. All patients were Han population in Northern China and were sporadic cases (i.e., not induced by radiation) ranging from 20 to 75 years old. The diagnosis of PTC was confirmed by pathologists based on the Revised American Thyroid Association Management Guidelines for Patients with Thyroid Nodules and Differentiated Thyroid Cancer [36]. The histologic type of PTC in our study is pure papillary carcinoma other than follicular variant of PTC, and metastasis of PTC was diagnosed as lymph node metastasis other than distant metastasis. Demographic data and clinical information were obtained through inquiry from the patients or from the medical records. Blood specimens were provided for genotyping. This project was approved by the ethics committee of the School of Public Health, Jilin University, and all subjects participated in the study with written informed consent.

### 2.2. Selection of SNPs in the ATM Gene

Four SNPs in the ATM gene were chosen on account of their potential function in the progress of cancer and the role they might play in altering activity of ATM kinase based on prior studies. The positions of rs664677 (human genome variation society (HGVS) name: NC_000011.9:g.108143182C>T), rs373759 (HGVS name: NC_000011.9:g.108220657C>T), rs4988099 (HGVS name: NC_000011.9:g.108194024A>G), and rs189037 (HGVS name: NC_000011.9:g.108093833G>A) in the gene were intron 20, intron 59, intron 45, and 5′ UTR of the promoter region, respectively. The minor allele frequencies (MAF) of the four SNPs were all more than 5%.

### 2.3. DNA Extraction

We collected 5 mL blood sample from every patient and stored the specimens at −20°C in nonanticoagulant, plexiglass tubes. Genomic DNA was extracted from peripheral blood lymphocytes by using a commercial DNA extraction kit (ClotBlood DNA kit, Cwbio, Beijing, China), according to the manufacturer's instructions. DNA concentration and purity were detected by ultraviolet spectrophotometer (Beckman, USA) with OD_260_/OD_280_ between 1.6 and 1.9 which we thought to be satisfactory.

### 2.4. SNP Genotyping

The primers for polymerase chain reaction (PCR) were designed by Assay Designer 3.1, showed in Table 1. PCR was performed on 384-well plate with well H11, H12, P11, and P12 as controls run without primer. The amplification system comprised 5 *μ*L mixture: HPLC-grade water 1.8 *μ*L, PCR Buffer 1x, MgCl_2_ 1.625 mM, dNTP Mix 500 *μ*M, Primer Mix 0.1 *μ*M, Hotstart Taq DNA Polymerase 1 U, and genomic DNA 1 *μ*L (10 ng). PCR cycle program was 15 min at 94°C, 45 cycles of 20 sec at 94°C, 30 sec at 56°C, and 1 min at 72°C, and the terminal extension was 3 min at 72°C. Genotypes of four SNPs were detected using the technique of Matrix-Assisted Laser Desorption/Ionization Time of Flight Mass Spectrometry (MALDI-TOF-MS) with the MassARRAY system (Sequenom, San, Diego, CA, USA). The genotyping rates for rs664677, rs373759, rs4988099, and rs189037 were 98.9%, 98.9%, 98.6%, and 99.2%, respectively.

### 2.5. Statistical Analysis

We analyzed the association between four SNPs in ATM and metastasis of PTC in males and females. Continuous covariates were presented using mean and standard deviation; for categorical variables, percentages were used instead. Association between genotypes and the gender-specific risk of metastasis was assessed by odds ratios (OR) and 95% confidence intervals (CI) under the unconditional logistic regression analysis adjusted by age and stage. For each SNP, risk of metastasis was estimated under different models of inheritance (codominant, dominant, recessive, overdominant, and multiplicative). Bonferroni correction was performed to reduce the type I error in multiple testing. The haplotype analysis was performed using the SNPStats program (http://bioinfo.iconcologia.net/SNPStats) [37] with the most frequent haplotype as the reference group. Departure from Hardy-Weinberg equilibrium (HWE) was evaluated using chi-square test. Pairwise linkage disequilibrium (LD) between SNPs was examined using *D*′ and *r* ^2^. All analyses were performed using SPSS 16.0 unless otherwise specified. Two-sided test with *P* value less than 0.05 was considered statistically significant.

## 3. Results

Table 2 showed the location, genotyping rate, test of HWE, minor allele, and MAF of the studied SNPs. All SNP genotype distributions were in HWE (*P* > 0.05) except for rs189037.

The study included 109 male patients and 249 female patients. The demographic and clinical characteristics of the participants enrolled in our study were summarized in Table 3. The mean ages of male and female patients were 43.23 and 42.53, respectively. 81.0% of males and 85.8% of females were diagnosed at an early tumor stage (stage I) with a few patients being diagnosed at latter stages. The proportion of patients with metastasis in males (47.6%) was higher than that in females (39.0%), and most patients, both males and females, were free of thyroid disease and family history of the disease. No significant difference in age, stage, metastasis, history of thyroid disease, and family history was found between male patients and female patients.

The distribution of genotypes for each SNP was summarized in Table 4. No significant difference in SNP genotype frequency was observed between patients with and without metastasis in both groups. For rs4988099, homozygote of the minor allele (GG) was absent in male group and only one GG genotype was detected in female patients without metastasis. In consideration of this, only distributions of AA and AG were compared for males in the following inheritance model analyses.

As seen from Table 5, in female patients, protective effect on PTC metastasis was observed for the rs189037 polymorphism. Under codominant and dominant models of inheritance, presence of minor A allele decreased the risk of metastasis compared with wild genotype GG (OR = 0.15, 95% CI 0.04–0.56, *P* = 0.01 for GA, OR = 0.08, 95% CI 0.01–0.74, *P* = 0.03 for AA, and OR = 0.49, 95% CI 0.25–0.98, *P* = 0.04 for GA + AA). In addition, the heterozygote GA also contributed to a decreased metastatic risk of PTC in the overdominant model (OR = 0.47, 95% CI 0.25–0.89, *P* = 0.02), while, under recessive and multiplicative models, AA genotype and increased number of A allele had no relationship with metastasis in female patients (*P* > 0.05). However, no association remained statistically significant after Bonferroni correction. These results collectively indicate that ATM rs189037 may be critical in metastasis of female PTCs and needs to be validated in studies with a larger sample size, while in male group no SNP significantly associated with metastasis of PTC was found.

For the low GG genotype frequency of rs4988099, LD examination and haplotype analysis were done among rs664677, rs373759, and rs189037 only. Strong linkage disequilibrium was observed between the three SNPs with each other (0.745 < *D*′ < 0.960, 0.495 < *r* ^2^ < 0.668).

Haplotype analysis was shown in Table 6. The two most common haplotypes CGG and TAA accounted for about 79% and 85% of all haplotypes in males and females, but no haplotype was found to be significantly associated with metastasis of PTC in both groups.

## 4. Discussion

SNP analysis is very common in the association study of genetic variation and its role in disease development. In our previous case-control study, we did not find significant tendency of risk alleles increasing from healthy controls to nonmetastatic PTCs, to metastatic PTCs using the trend chi-square test [38], while gender is an important factor in PTC because the incidence rate between males and females is distinct, so it is necessary to treat gender-specific PTC separately. In this stratification analysis, we demonstrated possible relevance of polymorphism in ATM rs189037 to metastatic risk of PTC in females though it became insignificant after Bonferroni correction. Further studies with larger sample size and different ethnic population would be needed to confirm our observation.

The polymorphism rs189037 is located at the 5′UTR of the promoter region of ATM gene. Studies have showed that polymorphisms in the promoter region of gene may be associated with specific phenotypes by changing the binding sites of transcription factors, which is important in gene expression [39]. The heterozygote of rs189037 has been observed to be significantly related to longevity probably in the way of regulating AP-2*α*, a transcription factor that participates in many important life processes [40]. We suppose that there is also one or more transcription factors whose binding sites are regulated by rs189037, and thus different genotypes may upregulate or downregulate the expression of ATM gene, which features in tumorigenesis and metastasis.

Some domestic studies found that ATM rs189037 A allele poorly predicted the outcome of lung cancer and was a risky biomarker of breast cancer in Taiwanese Females [41, 42]. Our results seem to be opposite to the above findings, and we guess that the role rs189037 plays in malignancy progression differs in the type of tumor and ethnicity. A study on rs189037 and nasopharyngeal carcinoma in Cantonese found no correlations even in gender-stratified analyses, providing evidence for our explanation [43].

It is useful to analyze the association stratified by gender for female gender is a well-known risk factor of thyroid cancer though the reason is unclear. A study using the Surveillance, Epidemiology, and End Results (SEER) 9 Registries Database identified that gender was an age-specific effect modifier for PTC incidence, and the female-to-male incidence rate declined consistently with increase in age [44]. The use of estrogen is one of the hypotheses that lead to higher thyroid cancer incidence rate in females, and Vivacqua et al. reported the possible molecular mechanism of thyroid cancer progression that involved estrogen [45]. However, whether estrogen functioned in the promotion of thyroid cancer metastasis needs to be further studied.

Our study has some limitations and should be interpreted with caution. Firstly, our study included 358 PTC patients, which is a relatively small sample size, and this might have influence on the statistical power. Secondly, although our inclusion criteria were made very strict, all patients were selected from hospital and selection bias is common in this kind of participants' recruitment. Thirdly, the exact mechanisms of ATM SNPs on progression and metastasis of PTC are unknown and further studies are needed in this respect. Nevertheless, our study provided a helpful clew to study the metastatic mechanism of PTC, especially for females.

## Figures and Tables

**Table 1:** Primers for polymerase chain reaction.

SNPs	Primer sequence (5′-3′)
rs664677	F: ACGTTGGATGCTCAGAAAACTCACTGAAAG
	R: ACGTTGGATGGGCATATTCCACATAATGAC

rs373759	F: ACGTTGGATGGTTAGCTTTTCTGCTGAGAG
	R: ACGTTGGATGTTCCCTCATACTCCTTCCTC

rs4988099	F: ACGTTGGATGAGTACATTGGCAGTACTTAC
	R: ACGTTGGATGCTCTTTTCAGCAGGATAATC

rs189037	F: ACGTTGGATGGCTAACGGAGAAAAGAAGCC
	R: ACGTTGGATGAGTAGTATCAACCGCGGC

F: forward; R: reverse.

**Table 2:** Information of the studied SNPs.

NCBI SNP ID	Location	Genotyping rate (%)	*P* (HWE)	Minor allele	MAF	
					In HAPMAP	In this study
rs664677	intron 20	98.9	0.199	T	0.388	0.418
rs373759	intron 59	98.9	0.092	A	0.305	0.381
rs4988099	intron 45	98.6	0.974	G	0.016	0.052
rs189037	5′-UTR	99.2	0.042	A	0.485	0.470

HWE: Hardy-Weinberg equilibrium; MAF: minor allele frequency; UTR: untranslated regions.

**Table 3:** Characteristics of the male and female patients (*n*, %).

Patient characteristics	Male (*n* = 109)	Female (*n* = 249)	*P*
Age^a^	43.23 ± 10.164	42.53 ± 9.035	0.517
Stage			0.723^b^
0-I	81 (81.0)	205 (85.8)	
II	7 (7.0)	15 (6.3)	
III	4 (4.0)	7 (2.9)	
IV	7 (7.0)	10 (4.2)	
Unknown	1 (1.0)	2 (0.8)	
Metastasis			0.145
Yes	49 (47.6)	89 (39.0)	
No	54 (52.4)	139 (61.0)	
History of thyroid disease			0.526
Yes	8 (7.4)	14 (5.6)	
No	100 (92.6)	234 (94.4)	
Family history			0.902
Yes	7 (6.6)	17 (7.0)	
No	99 (93.4)	227 (93.0)	

^a^Mean ± standard deviation (SD).

^b^Fisher's exact test.

**Table 4:** Distribution of genotype frequency by study groups.

Genotype	Male		*P*	Female		*P*
	Metastasis (+)	Metastasis (−)		Metastasis (+)	Metastasis (−)	
rs664677			0.993			0.088
CC	15 (31.3)	16 (30.2)		34 (38.6)	44 (31.9)	
CT	24 (50.0)	27 (50.9)		38 (43.2)	79 (57.2)	
TT	9 (18.8)	10 (18.9)		16 (18.2)	15 (10.9)	
rs373759			0.359			0.249
GG	11 (22.9)	19 (35.8)		35 (39.8)	53 (38.4)	
GA	31 (64.6)	29 (54.7)		38 (43.2)	71 (51.4)	
AA	6 (12.5)	5 (9.4)		15 (17.0)	14 (10.1)	
rs4988099			0.493^a^			0.406
AA	45 (93.8)	47 (88.7)		81 (92.0)	119 (86.9)	
AG	3 (6.3)	6 (11.3)		7 (8.0)	17 (12.4)	
GG	0 (0.0)	0 (0.0)		0 (0.0)	1 (0.7)	
rs189037			0.317			0.126
GG	7 (14.3)	12 (22.6)		32 (36.4)	36 (26.1)	
GA	33 (67.3)	28 (52.8)		39 (44.3)	80 (58.0)	
AA	9 (18.4)	13 (24.5)		17 (19.3)	22 (15.9)	

^a^Fisher's exact test.

**Table 5:** OR (95% CI) for metastasis of PTC by ATM gene polymorphisms according to different models of inheritance (adjusted by age and stage). *P* < 0.05 in bold.

SNP, genotype	Male		Female	
	OR (95% CI)	P	OR (95% CI)	*P*
rs664677				
CC^a^	1.00		1.00	
CT	0.36 (0.04–3.41)	0.54	4.32 (0.54–34.27)	0.95
TT	0.63 (0.14–2.73)	0.38	1.03 (0.40–2.66)	0.17
CT + TT versus CC^b^	0.98 (0.35–2.75)	0.97	0.74 (0.39–1.41)	0.36
TT versus CC + CT^c^	0.75 (0.21–2.64)	0.65	2.03 (0.86–4.82)	0.11
CT versus CC + TT^d^	1.17 (0.44–3.12)	0.75	0.54 (0.29–1.00)	0.05
Risk per T allele^e^	0.91 (0.46–1.80)	0.78	1.05 (0.66–1.67)	0.84
rs373759				
GG^a^	1.00		1.00	
GA	1.94 (0.43–8.73)	0.39	3.11 (0.98–9.88)	0.06
AA	4.67 (0.34–65.00)	0.25	3.01 (0.34–26.90)	0.32
GA + AA versus GG^b^	1.81 (0.62–5.31)	0.27	0.98 (0.52–1.84)	0.96
AA versus GG + GA^c^	0.81 (0.15–4.24)	0.80	1.66 (0.68–4.07)	0.27
GA versus GG + AA^d^	1.83 (0.67–4.99)	0.24	0.78 (0.42–1.44)	0.43
Risk per A allele^e^	1.34 (0.60–2.98)	0.48	1.13 (0.71–1.78)	0.61
rs4988099				
AA^a^	1.00^f^		1.00	
AG	0.73 (0.13–3.97)	0.71	0.52 (0.17–1.57)	0.24
AG + GG versus AA^b^	—	—	0.68 (0.24–1.94)	0.46
AG versus AA + GG^c^	—	—	0.70 (0.25–2.00)	0.50
Risk per G allele^e^	—	—	0.68 (0.24–1.88)	0.44
rs189037				
GG^a^	1.00		1.00	
GA	1.58 (0.23–10.87)	0.64	**0.15 (0.04–0.56)**	**0.01**
AA	0.78 (0.05–13.51)	0.87	**0.08 (0.01–0.74)**	**0.03**
GA + AA versus GG^b^	1.87 (0.52–6.74)	0.33	**0.49 (0.25–0.98)**	**0.04**
AA versus GG + GA^c^	0.55 (0.14–2.09)	0.37	1.29 (0.58–2.86)	0.53
GA versus GG + AA^d^	2.18 (0.78–6.11)	0.13	**0.47 (0.25–0.89)**	**0.02**
Risk per A allele^e^	1.03 (0.47–2.25)	0.95	0.79 (0.49–1.25)	0.31

^a^Codominant model (wild homozygote serves as the reference).

^b^Dominant model (combined heterozygote and homozygote for the minor allele versus wild homozygote).

^c^Recessive model (minor allele homozygote versus combined heterozygote and homozygote for the wild allele).

^d^Overdominant model (heterozygote versus combined homozygote for the wild and minor alleles).

^e^Multiplicative model (uses allele frequencies).

^f^Just gives OR (95% CI) and *P*-value for heterozygote versus wild homozygote (no GG genotype for males).

**Table 6:** Associations between ATM haplotypes and risk of metastasis (adjusted by age and stage).

Haplotype	SNP^a^			Frequency			OR (95% CI)	*P*
	1	2	3	Total	Metastasis (−)	Metastasis (+)		
Male								
1	C	G	G	0.4512	0.4699	0.4324	1.00	—
2	T	A	A	0.3387	0.3217	0.3600	1.14 (0.47–2.75)	0.78
3	T	G	A	0.0794	0.1010	0.0532	0.43 (0.09–2.13)	0.31
4	C	A	A	0.0658	0.0462	0.0852	2.42 (0.43–13.79)	0.32
5	C	G	A	0.0308	0.0405	0.0220	1.39 (0.14–13.42)	0.78
6	T	G	G	0.0223	0.0207	0.0240	2.97 (0.24–37.16)	0.40
Rare^b^	—	—	—	0.0118	0.0000	0.0232	—	—
Female								
1	C	G	G	0.5330	0.5228	0.5494	1.00	—
2	T	A	A	0.3142	0.2983	0.3386	1.00 (0.60–1.66)	0.99
3	T	G	A	0.0532	0.0687	0.0295	0.30 (0.08–1.10)	0.07
4	C	A	A	0.0460	0.0529	0.0361	0.54 (0.16–1.84)	0.32
5	T	G	G	0.0241	0.0240	0.0241	1.45 (0.38–5.51)	0.58
6	C	G	A	0.0224	0.0294	0.0106	0.35 (0.06–2.13)	0.26
Rare^b^	—	—	—	0.0070	0.0039	0.0117	3.03 (0.18–52.22)	0.45

^a^SNP are as follows: 1: rs664677; 2: rs373759; 3: rs189037.

^b^Rare: haplotypes with frequencies <0.01.

## References

[1] Xing M., Alzahrani A. S., Carson K. A. (2013). Association between BRAF V600E mutation and mortality in patients with papillary thyroid cancer. *The Journal of the American Medical Association*.

[2] Choi J. Y., Hwang B. H., Jung K. C., Min H. S., Koo D. H., Youn Y.-K., Lee K. E. (2013). Clinical significance of microscopic anaplastic focus in papillary thyroid carcinoma. *Surgery*.

[3] Cipollini M., Pastor S., Gemignani F., Castell J., Garritano S., Bonotti A., Biarnés J., Figlioli G., Romei C., Marcos R., Cristaudo A., Elisei R., Landi S., Velázquez A. (2013). TPO genetic variants and risk of differentiated thyroid carcinoma in two European populations. *International Journal of Cancer*.

[4] Brenner A. V., Neta G., Sturgis E. M., Pfeiffer R. M., Hutchinson A., Yeager M., Xu L., Zhou C., Wheeler W., Tucker M. A., Chanock S. J., Sigurdson A. J. (2013). Common single nucleotide polymorphisms in genes related to immune
function and risk of papillary thyroid cancer. *PLoS ONE*.

[5] Sturgis E. M., Li G. (2009). Molecular epidemiology of papillary thyroid cancer: in search of common genetic associations. *Thyroid*.

[6] Xu L., Li G., Wei Q., El-Naggar A. K., Sturgis E. M. (2012). Family history of cancer and risk of sporadic differentiated thyroid carcinoma. *Cancer*.

[7] Rahbari R., Zhang L., Kebebew E. (2010). Thyroid cancer gender disparity. *Future Oncology*.

[8] Ron E., Lubin J. H., Shore R. E., Mabuchi K., Modan B., Pottern L. M., Schneider A. B., Tucker M. A., Boice J. D. (1995). Thyroid cancer after exposure to external radiation: a pooled analysis of seven studies. *Radiation Research*.

[9] Sinnott B., Ron E., Schneider A. B. (2010). Exposing the thyroid to radiation: a review of its current extent, risks, and implications. *Endocrine Reviews*.

[10] Enewold L., Zhu K., Ron E., Marrogi A. J., Stojadinovic A., Peoples G. E., Devesa S. S. (2009). Rising thyroid cancer incidence in the United States by demographic and tumor characteristics, 1980–2005. *Cancer Epidemiology Biomarkers & Prevention*.

[11] Jung K. W., Park S., Konget H. J. (2011). Cancer statistics in Korea: incidence, mortality, survival, and prevalence in 2008. *Cancer Research and Treatment*.

[12] Sipos J. A., Mazzaferri E. L. (2010). Thyroid cancer epidemiology and prognostic variables. *Clinical Oncology*.

[13] Hundahl S. A., Fleming I. D., Fremgen A. M., Menck H. R. (1998). A National Cancer Data Base report on 53,856 cases of thyroid carcinoma treated in the US, 1985–1995. *Cancer*.

[14] Tuttle R. M., Ball D. W., Byrdet D. (2010). Thyroid carcinoma. *Journal of the National Comprehensive Cancer Network*.

[15] Landa I., Ruiz-Llorente S., Montero-Conde C., Inglada-Pérez L., Schiavi F., Leskelä S., Pita G., Milne R., Maravall J., Ramos I., Andía V., Rodríguez-Poyo P., Jara-Albarrán A., Meoro A., Del Peso C., Arribas L., Iglesias P., Caballero J., Serrano J., Picó A., Pomares F., Giménez G., López-Mondéjar P., Castello R., Merante-Boschin I., Pelizzo M.-R., Mauricio D., Opocher G., Rodríguez-Antona C., González-Neira A., Matías-Guiu X., Santisteban P., Robledo M. (2009). The variant rs1867277 in FOXE1 gene confers thyroid cancer susceptibility through the recruitment of USF1/USF2 transcription factors. *PLoS Genetics*.

[16] Xing M. (2007). BRAF mutation in papillary thyroid cancer: pathogenic role, molecular bases, and clinical implications. *Endocrine Reviews*.

[17] Basolo F., Giannini R., Faviana P., Fontanini G., Malizia A. P., Ugolini C., Elisei R., Miccoli P., Toniolo A. (2004). Thyroid papillary carcinoma: preliminary evidence for a germ-line single nucleotide polymorphism in the Fas gene. *Journal of Endocrinology*.

[18] Gudmundsson J., Sulem P., Gudbjartsson D. F., Jonasson J. G., Sigurdsson A., Bergthorsson J. T., He H., Blondal T., Geller F., Jakobsdottir M., Magnusdottir D. N., Matthiasdottir S., Stacey S. N., Skarphedinsson O. B., Helgadottir H., Li W., Nagy R., Aguillo E., Faure E., Prats E., Saez B., Martinez M., Eyjolfsson G. I., Bjornsdottir U. S., Holm H., Kristjansson K., Frigge M. L., Kristvinsson H., Gulcher J. R., Jonsson T., Rafnar T., Hjartarsson H., Mayordomo J. I., De La Chapelle A., Hrafnkelsson J., Thorsteinsdottir U., Kong A., Stefansson K. (2009). Common variants on 9q22.33 and 14q13.3 predispose to thyroid cancer in European populations. *Nature Genetics*.

[19] Ricaud L., Proux C., Renou J.-P., Pichon O., Fochesato S., Ortet P., Montané M.-H. (2007). ATM-mediated transcriptional and developmental responses to *γ*-rays in *Arabidopsis*. *PLoS ONE*.

[20] Savitsky K., Bar-Shira A., Gilad S., Rotman G., Ziv Y., Vanagaite L., Tagle D. A., Smith S., Uziel T., Sfez S., Ashkenazi M., Pecker I., Frydman M., Harnik R., Patanjali S. R., Simmons A., Clines G. A., Sartiel A., Gatti R. A., Chessa L., Sanal O., Lavin M. F., Jaspers N. G. J., Taylor A. M. R., Arlett C. F., Miki T., Weissman S. M., Lovett M., Collins F. S., Shiloh Y. (1995). A single ataxia telangiectasia gene with a product similar to PI-3 kinase. *Science*.

[21] Lavin M. F., Scott S., Gueven N., Kozlov S., Peng C., Chen P. (2004). Functional consequences of sequence alterations in the ATM gene. *DNA Repair*.

[22] Bakkenist C. J., Kastan M. B. (2003). DNA damage activates ATM through intermolecular autophosphorylation and dimer dissociation. *Nature*.

[23] Xu L., Morari E. C., Wei Q., Sturgis E. M., Ward L. S. (2012). Functional variations in the ATM gene and susceptibility to differentiated thyroid carcinoma. *Journal of Clinical Endocrinology and Metabolism*.

[24] Lavin M. F. (2008). Ataxia-telangiectasia: from a rare disorder to a paradigm for cell signalling and cancer (*Nature Reviews Molecular Cell Biology*, vol. 9, pp. 759–769, 2008). *Nature Reviews Molecular Cell Biology*.

[25] Flanagan J. M., Munoz-Alegre M., Henderson S., Tang T., Sun P., Johnson N., Fletcher O., dos Santos Silva I., Peto J., Boshoff C., Narod S., Petronis A. (2009). Gene-body hypermethylation of ATM in peripheral blood DNA of bilateral breast cancer patients. *Human Molecular Genetics*.

[26] Kim J. H., Kim H., Lee K. Y., Choe K.-H., Ryu J.-S., Yoon H. I., Sung S. W., Yoo K.-Y., Hong Y.-C. (2006). Genetic polymorphisms of ataxia telangiectasia mutated affect lung cancer risk. *Human Molecular Genetics*.

[27] Akulevich N. M., Saenko V. A., Rogounovitch T. I., Drozd V. M., Lushnikov E. F., Ivanov V. K., Mitsutake N., Kominami R., Yamashita S. (2009). Polymorphisms of DNA damage response genes in radiation-related and sporadic papillary thyroid carcinoma. *Endocrine-Related Cancer*.

[28] Meyer A., Wilhelm B., Dörk T., Bremer M., Baumann R., Karstens J. H., Machtens S. (2007). ATM missense variant P1054R predisposes to prostate cancer. *Radiotherapy and Oncology*.

[29] Rudd M. F., Sellick G. S., Webb E. L., Catovsky D., Houlston R. S. (2006). Variants in the ATM-BRCA2-CHEK2 axis predispose to chronic lymphocytic leukemia. *Blood*.

[30] He Y., Chen Q., Li B. (2008). ATM in oral carcinogenesis: association with clinicopathological features. *Journal of Cancer Research and Clinical Oncology*.

[31] Kang B., Guo R.-F., Tan X.-H., Zhao M., Tang Z.-B., Lu Y.-Y. (2008). Expression status of ataxia-telangiectasia-mutated gene correlated with prognosis in advanced gastric cancer. *Mutation Research—Fundamental and Molecular Mechanisms of Mutagenesis*.

[32] Sun M., Guo X., Qian X. (2012). Activation of the ATM-snail pathway promotes breast cancer metastasis. *Journal of Molecular Cell Biology*.

[33] Lee J., Sung C. O., Lee E. J., Do I.-G., Kim H.-C., Yoon S. H., Lee W. Y., Chun H. K., Kim K.-M., Park Y. S. (2012). Metastasis of neuroendocrine tumors are characterized by increased cell proliferation and reduced expression of the atm gene. *PLoS ONE*.

[34] Lang B. H.-H., Lo C.-Y., Chan W.-F., Lam K.-Y., Wan K.-Y. (2007). Prognostic factors in papillary and follicular thyroid carcinoma: their implications for cancer staging. *Annals of Surgical Oncology*.

[35] Mazzaferri E. L., Kloos R. T. (2001). Current approaches to primary therapy for papillary and follicular thyroid cancer. *The Journal of Clinical Endocrinology & Metabolism*.

[36] Cooper D. S., Doherty G. M., Haugen B. R., Kloos R. T., Lee S. L., Mandel S. J., Mazzaferri E. L., McIver B., Pacini F., Schlumberger M., Sherman S. I., Steward D. L., Tuttle R. M. (2009). Revised American thyroid association management guidelines for patients with thyroid nodules and differentiated thyroid cancer. *Thyroid*.

[37] Solé X., Guinó E., Valls J., Iniesta R., Moreno V. (2006). SNPStats: a web tool for the analysis of association studies. *Bioinformatics*.

[38] Gu Y., Yu Y., Ai L., Shi J., Liu X., Sun H., Liu Y. (2014). Association of the ATM gene polymorphisms with papillary thyroid cancer. *Endocrine*.

[39] Sun T., Gao Y., Tan W., Ma S., Shi Y., Yao J., Guo Y., Yang M., Zhang X., Zhang Q., Zeng C., Lin D. (2007). A six-nucleotide insertion-deletion polymorphism in the CASP8 promoter is associated with susceptibility to multiple cancers. *Nature Genetics*.

[40] Chen T., Dong B., Lu Z. (2010). A functional single nucleotide polymorphism in promoter of ATM is associated with longevity. *Mechanisms of Ageing and Development*.

[41] Dong J., Hu Z., Shu Y., Pan S., Chen W., Wang Y., Hu L., Jiang Y., Dai J., Ma H., Jin G., Shen H. (2012). Potentially functional polymorphisms in DNA repair genes and non-small-cell lung cancer survival: a pathway-based analysis. *Molecular Carcinogenesis*.

[42] Wang H.-C., Chang W.-S., Tsai R. U.-Y., Tsai C.-W., Liu L.-C., Su C.-H., Cheng H.-N. I., Tsou Y.-A. N., Sun S.-S., Lin C.-C., Bau D. A.-T. (2010). Association between ataxia telangiectasia mutated gene polymorphisms and breast cancer in taiwanese females. *Anticancer Research*.

[43] Wang H.-M., Shi Y.-S., Li Q.-S., Liu Y., Zheng X.-K. (2011). Association between single nucleotide polymorphism locus rs189037 in the promoter of ATM gene and nasopharyngeal carcinoma susceptibility in Cantonese. *Journal of Southern Medical University*.

[44] Kilfoy B. A., Devesa S. S., Ward M. H., Zhang Y., Rosenberg P. S., Holford T. R., Anderson W. F. (2009). Gender is an age-specific effect modifier for papillary cancers of the thyroid gland. *Cancer Epidemiology Biomarkers and Prevention*.

[45] Vivacqua A., Bonofiglio D., Albanito L., Madeo A., Rago V., Carpino A., Musti A. M., Picard D., Andò S., Maggiolini M. (2006). 17*β*-Estradiol, genistein, and 4-hydroxytamoxifen induce the proliferation of thyroid cancer cells through the G protein-coupled receptor GPR30. *Molecular Pharmacology*.

